# Rituximab 500 mg 6-monthly infusions is an option in maintenance therapy of ANCA-associated vasculitis

**DOI:** 10.1093/rap/rkab039

**Published:** 2021-07-16

**Authors:** Ruchika Goel, Matthew Morgan, Dimitrios Chanouzas, Joshua Caplan, Sarah Logan, Lorraine Harper

**Affiliations:** 1 Institute of Clinical Sciences, Centre for Translational Inflammation Research, University of Birmingham Research Laboratories, Queen Elizabeth Hospital, Birmingham, UK; 2 Department of Clinical Immunology and Rheumatology, Christian Medical College, Vellore, India; 3 Hull York Medical School, University of Hull, Hull; 4 Renal Medicine, University Hospitals Birmingham NHS Foundation Trust; 5 Sandwell and West Birmingham Hospitals NHS Trust, Birmingham, UK

Key message

•Reduced dose rituximab appears to be an effective option as maintenance therapy in ANCA-associated vasculitis.

Dear Editor, Rituximab is a promising option for maintenance of remission in ANCA-associated vasculitis (AAV) , particularly in patients with relapsing disease [1–3]. The optimal dose, frequency and duration of maintenance rituximab remain unclear. The doses reported from longitudinal studies and clinical trials range from 1000 mg every 4 months to as low as 500 mg every 6 months [1, 4, 5]. Although the latter regimen was superior to AZA in maintenance of remission, the dose recommended by experts in the UK remains either 1000 or 500 mg every 6 months for 2 years [6]. The uncertainty in dosing might be attributable to lack of direct comparison between different dosing regimens. This retrospective service evaluation aimed to determine the effectiveness and safety of reduced dosage of rituximab (500 mg every 6 months) in maintaining remission in patients with AAV compared with higher-dose regimens .

Medical records of patients with AAV attending the Vasculitis clinics between November 2002 and April 2020 at University Hospitals Birmingham NHS Foundation Trust (UHB NHSFT) UK were reviewed. Demographics, treatment details and disease activity of patients receiving rituximab for maintaining remission were recorded.

Patients were included in the study if they received a minimum of: 2 years of maintenance treatment with either 2000 mg/year or 1000 mg/6 months; or two 1000 mg infusions followed by at least two 500 mg infusion over 2 years; or three 500 mg infusions over 18 months.

All patients had a minimum follow-up of 6 months after their initial maintenance treatment period.

Relapses were defined as a birmingham vasculitis activity score (BVAS) of more than one. The infections were recorded from the clinician’s notes, and the infection rate was calculated. The results are reported in a descriptive manner, with frequencies reported as the median and interquartile range. The χ^2^ test was used for comparison among the multiple groups. The study was registered and approved by Clinical Audit Registration unit of UHB NHSFT.

From 104 patients receiving rituximab maintenance therapy, 62 patients satisfied inclusion criteria. The patients were categorized into four groups based on the dose of maintenance rituximab as follows: 2000 mg/year regimen (regimen A); 1000 mg/6 months infusion (regimen B); 1000 mg/6 months followed by 500 mg/6 months (regimen C); and upfront 500 mg/6 months rituximab (regimen D). Sixteen patients received regimen A, and 21 patients received regimen B, which included 8 patients who were switched to this regimen after receiving ≥2 years of regimen A. Reduced dose rituximab (500 mg 6-monthly) was prescribed for 33 patients, which included 27 and 6 patients given regimen C and D, respectively (Supplementary Fig. S1, available at *Rheumatology Advances in Practice* online). Patients who were given regimen C received a median of 4 (3–6) 6-monthly infusions of 1000 mg rituximab followed by 4 (3–4) 6-monthly infusions of 500 mg, and patients in regimen D received 3.5 (3–4.25) infusions of 500 mg/6 months rituximab (Table 1). The demographics and clinical details of patients are presented in Supplementary Table S1, available at *Rheumatology Advances in Practice* online .

**Table 1:** Treatment and outcome details of patients with ANCA-associated vasculitis on maintenance rituximab

Parameter	2000 mg every 12 months, *n* = 16	1000 mg every 6 months, *n* = 21	1000 mg/6 months followed by 500 mg/6 months, *n* = 27^a^		500 mg/6 months as first-line therapy, *n* = 6
	Regimen A	Regimen B	Regimen C		Regimen D
			Phase 1 (1000 mg)	Phase 2 (500 mg)	
Disease duration before initiating maintenance rituximab, median (IQR), months	60 (14–90)	84 (18–132)	72 (36–144)	104 (60–156)	12 (10–159)
Prior rituximab exposure, g^b^	0 0	4 (2–6)	2 (2–4.5)	4 (3–6)	2 (1.5–5.25)
Indication					
Relapsing disease	12	16	21		2
Refractory disease	2	3	3		3
Contraindication to non-biologic immunosuppression	2	6	10		2
ANCA positive	16	19	27		5
Anti-PR3	15	15	15		2
Anti-MPO	1	4	10		3
Anti PR3 and anti-MPO	0	0	1		0
Negative	0	2	1		1
Infusions, median (IQR), *n*	6 (5–8)	5 (4–7)	4 (3–6)	4 (3–4)	3.5 (3–4.25)
Duration of follow-up on maintenance rituximab, median (IQR), years	5.4 (4.1–6.8)	2.2 (1.5–2.8)	1.5 (1– 2.5)	1.2 (1.1–1.9)	1.3 (1.0–1.7)
Concomitant IS, no. of patients	8	3	3	0	0
Outcome					
Relapse, no. of patients	14	0	3	1	0
Major relapse, no. of patients	10	0	1	0	0
Minor relapse, no. of patients	4	0	2	1	0
Total no. of relapses	43	0	3	1	0
Time to first relapse, median (IQR), months	12 (9–13)	–	38 (14–54)	3 (–)	–
Adverse events					
Infection, no. of patients	13	10	18	10	3
Episodes of infection, total *n*	54	18	36	14	4
Episodes of serious infection, no. of patients^**^	11 (8)	1 (1)	0	1(1)	0
Hypogammaglobulinemia, *n* (tested)**^c^					
Persistent	1 (16)	4 (21)	9 (27)	9 (27)	4 (6)
New/worsening	8 (16)	5 (21)	4 (27)	5 (27)	1 (6)

a Also includes patients who were included in the 1000 mg/6 months group and were subsequently switched to 500 mg/6 months regimen. ^b^Includes rituximab doses given for induction of remission. ^c^Defined as new-onset reduction in immunoglobulin (IgG) to <6 g/dl or any further decrease in IgG levels in patients with baseline IgG of <6 g/dl at the first maintenance dose. **P < 0.01. IQR: interquartile range; IS: immunosuppressant.

During follow-up, the proportion of patients who relapsed and required escalation of CS dose was significantly higher in regimen A (43 relapses in 14 patients) compared with no relapses in regimens B and D and four relapses in 21 patients in regimen C, *P* < 0.01 (Table 1). Among four relapses observed in patients given regimen C, three were observed while receiving 1000 mg/6-monthly infusions, whereas one patient relapsed while receiving extended maintenance 500 mg/6-monthly rituximab. None of the patients given regimen B or regimen D relapsed. The median time to relapse was 38 (14–54) months for patients receiving regimen C and 12 (8.75–13) months for those receiving regimen A.

The rate of infection was 0.47 (95% CI: 0.27, 0.77) per patient per year in patients receiving regimen A, 0 (0–0.52) in patients receiving regimen B, 0 (0–1.07) in patients receiving regimen C and 0.24 (0–0.98) in patients regimen D. The rate of serious infections requiring hospitalizations was 0.04 (0.00–0.18) per patient per year (11 episodes in 8 patients) in patients on regimen A, 0.00 (0.00) each in in patients receiving regimen B (1 episode in 1 patient) and those receiving group C (1 episode in 1 patient). None of the patients receiving regimen D reported serious infection. Owing to the retrospective nature of the study, some infections might have gone unreported, adding to the limitations of the study.

New-onset/worsening hypogammaglobulinemia, defined as new-onset reduction in immunoglobulin G (IgG) to <6 g/dl or any further decrease in IgG levels in patients with baseline IgG of <6 g/dl at the first maintenance dose, was observed in 50% of patients given regimen A compared with 18.8%, 18.5% and 16.7% on regimen B, C and D rituximab, respectively (see Table 1). Previous use of CYC has been associated with an increased risk of hypogammaglobulinaemia after rituximab therapy [7]. However, this does not explain the difference in hypogammaglobulinaemia between regimens A and B (Table 1).

Although this is a small retrospective study, 500 mg 6-monthly infusions appear to be an effective and safe option in maintenance of remission in AAV, either after previous 1 g 6-monthly or 500 mg 6-monthly from start of maintenance rituximab therapy. This study provides real-world data to reiterate the effectiveness of reduced rituximab dose. Studies with longer follow-up are required to confirm the observations.

## Supplementary Material

rkab039_Supplementary_Data
