# Supplementary material for: Rituximab 500 mg 6-monthly infusions is an option in maintenance therapy of ANCA-associated vasculitis
Source: Rheumatol Adv Pract. 2021 Jul 16;5(2):rkab039. doi: 10.1093/rap/rkab039 (PMC8496757; doi:10.1093/rap/rkab039)
Supplement: rkab039_Supplementary_Data [file rkab039_supplementary_data.docx]

Supplementary Figure S1: Flow chart of patients included in the study


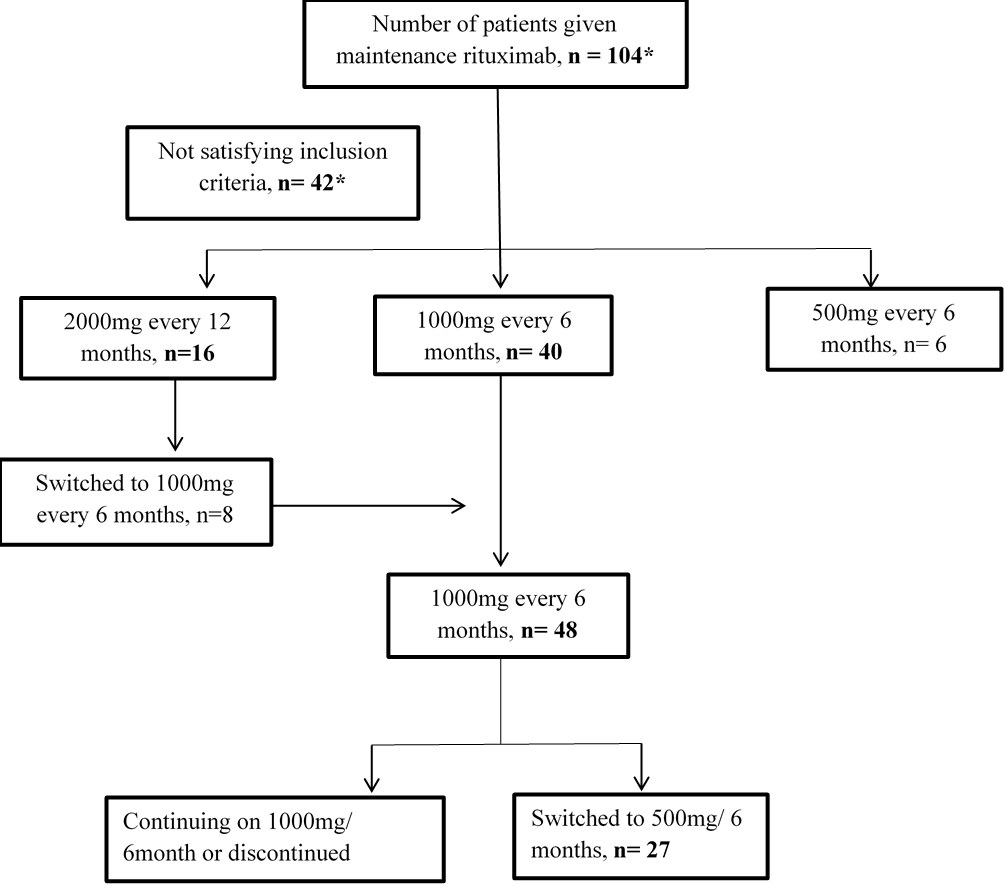


Supplementary Table S1: Baseline demography and clinical details of the patients treated with Rituximab

| **Dosage regime** | **2000mg/ 12months, n=16**  **Regime A** | **1000mg/ 6 months, n= 21**  **Regime B** | **Reduced dose (500mg every 6 months), n=33** | |
| --- | --- | --- | --- | --- |
|  |  |  | **Following 1000mg/ 6 monthly Regime C** | **As first line therapy Regime D** |
| Number of patients | 16 | 21 | 27 | 6 |
| Age at diagnosis in years (median, IQR) | 48 (31.5-56.8) | 49 (32.5-57.0) | 55 (43.0-65.0) | 70.3 (52.3-79.0) |
| Gender (M:F) | 9:7 | 12:9 | 14:13 | 3:3 |
| Diagnosis n (%)  GPA/MPA/EGPA/unclassified | 15/1/0/0  (93.7/ 6.3/0/0) | 15/5/1/0  (71.4/ 23.8/4.7) | 16/6/1/3  (59.3/ 22.2/3.7/11.1) | 3/1/2  (50.0/16.7/33.3) |
| Organ involvement, n (%)  Eye  ENT (ear)  ENT (nose/throat)  CNS  Pulmonary  Renal  Skin  Systemic features | 7 (50.0)  1 (6.3)  13 (92.9)  6 (42.9)  7 (50.0)  7 (50.0)  5 (35.7)  1 (6.3) | 6 (31.6)  7 (35.0)  13 (65.0)  2 (10.0)  15 (71.4)  14 (66.7)  5 (25)  13 (65) | 7 (28.0)  6 (26.0)  14 (56.0)  44 (11.0)  14 (56.0)  19 (70.3)  5 (20)  17 (68) | 1(16.7)  2 (33.5)  3 (50.0)  1 (16.7)  3 (50.0)  6 (100)  1 (16.7)  4 (66.6) |
| Median BVAS (at presentation) | 7 (3.3-8.8) | 14 (10.8-22) | 15 (11.5-20.5) | 16 (7.5-19.5) |
| Prior immunosuppression, n (%)  Cyclophosphamide  Azathioprine  Mycophenolate (induction)  Mycophenolate (maintenance)  MTX  Plasmapheresis  Infliximab | 12 (75.0)  7 (43.8)  1 (6.3)  6 (37.5)  3 (18.8)  1 (6.3)  1 (6.3%) | 21 (100)  5 (23.8)  3 (14.3)  9 (42.9)  4 (19.0)  3 (14.3)  2 (8.5) | 16 (59.3)  18 (66.7)  5 (18.5)  11 (40.7)  3 (11.1)  10 (37.0)  1 (3.7) | 3 (50.0)  2 (33.3)  0 (0)  1 (16.6)  0 (0)  1 (16.6)  0 (0) |

GPA granulomatous polyangiitis, MPA microscopic Polyangitis, EGPA Eosinophilic granulomatous polyangiitis,
